# Do Parents Perceive Practitioners to Have a Specific Role in Change? A Longitudinal Study Following Participation in an Evidence-Based Program

**DOI:** 10.3390/ijerph19159100

**Published:** 2022-07-26

**Authors:** Sara M. Leitão, Marco Pereira, Rita V. Santos, Maria Filomena Gaspar, Maria João Seabra-Santos

**Affiliations:** 1 Center for Research in Neuropsychology and Cognitive and Behavioral Intervention, Faculty of Psychology and Educational Sciences, University of Coimbra, 3000-115 Coimbra, Portugal; marcopereira@fpce.uc.pt (M.P.); seabramj@fpce.uc.pt (M.J.S.-S.); 2 Faculty of Psychology and Educational Sciences, University of Coimbra, 3000-115 Coimbra, Portugal; riitasantos4@gmail.com; 3 Centre for Social Studies, Faculty of Psychology and Educational Sciences, University of Coimbra, 3000-995 Coimbra, Portugal; ninigaspar@fpce.uc.pt

**Keywords:** practitioner, skills, parenting, change, evidence-based

## Abstract

Little attention has been given to the role of practitioners in evidence-based parenting programs and to the evaluation that parents make of their importance in the process of change. This study aims to explore the role that parents assign to the facilitators of the Incredible Years (IY) program in enabling long-term life changes, as well as the association between parents’ evaluation of the practitioners’ skills and specific changes perceived after the intervention. In this longitudinal study, we applied 1 survey to 80 community parents who had participated in an IY group 2 years before, and we retrieved archival data to assess changes in parents’ ratings of sense of competence and in children’s behaviors immediately after the end of the intervention. Two years after the intervention, parents perceived significant improvements, especially in their parenting and their children’s behaviors, and they recognized that their IY practitioners had played a significant role in these life changes. Parents who attributed a greater role to the practitioners’ skills reported a greater number of improvements in parental sense of competence and in children’s behaviors. The practitioners’ skills relating more broadly to these specific changes are the practitioners’ sensitivity and flexibility towards parents’ needs and the practitioners’ ability to clearly share knowledge with parents. The practitioner’s assigned role when implementing an evidence-based parenting program seems to go far beyond the mere conveyance of the program’s specific contents and methods and deserves to be researched further.

## 1. Introduction

Parent-based interventions are one of the most widely researched effective interventions for the prevention and treatment of child and youth behavior problems [1,2]. The effectiveness of parenting interventions in reducing child conduct problems and in improving parenting practices has been consistently demonstrated in several reviews [3,4]. Positive effects are present not only immediately after the intervention but also in the long term [5,6], with a meta-analysis suggesting that the effects of an intervention were present up to three years following the intervention [6]. There has also been some evidence of the secondary effects of these interventions on variables that were not directly targeted by the programs, especially in reducing parenting stress, improving perceived parenting competence and self-efficacy, and affecting other interpersonal relationships, such as the marital relationship and overall quality of family life [4,7,8,9].

In recent decades, governments and local authorities have required the widespread dissemination of effective parenting interventions, and many parenting programs have been developed and have proliferated in naturalistic and community settings [10]. Generally, these interventions aim to promote the adoption of an optimal parenting style and are in line with recent research emphasizing the importance of parental warmth for the positive development of children [11]. Parenting programs are generally structured, short-term interventions provided individually or in a group format by a range of professionals [12]. Although these programs are usually manualized and supported by standardized procedures and materials, it is known that large-scale service-based replications are highly susceptible to program drift [13]. Moreover, the fact that these programs are delivered by professionals from different backgrounds, such as psychologists, nurses, counselors, or social or community workers [12], may increase the variability in the practitioners’ skills and quality of delivery, particularly in community contexts, where resources for the training, supervision, and retention of skilled staff are often more tenuous.

Factors related to the practitioner implementing the intervention have long been considered a key variable among the process skills needed to deliver any intervention effectively, as it is the manner in which the content is delivered that contributes to the development of the therapeutic relationship and that guides positive participant outcomes [14]. However, in the field of evidence-based parenting programs, the study of the practitioner’s role in the outcomes tends not to be a frequent research focus [15,16], especially if compared with the more common existing research on the role of the intervention characteristics, such as program contents and delivery methods [17,18,19]. Different titles have been used in the literature to refer to the professional who is implementing the parenting program (e.g., facilitator, deliverer, group leader, practitioner, therapist). In the present article, our preference is to use the term “practitioner” as we consider it more neutral and more adequate given the diversity of application contexts of parenting programs. However, when describing other studies’ results, we will maintain the designations used by the original authors out of respect for their stances. A recent systematic review of 24 quantitative studies demonstrated that despite the fact that research on this topic is weak and sparse, positive intervention outcomes were consistently associated with practitioner-related factors in different evidence-based parenting programs [15]. Specifically, this review presented evidence that changes in parenting practices were consistently related to the practitioners’ contributions to the parent–therapist alliance, their fidelity to the intervention and their specific in-session actions reflecting both interpersonal and more directive skills. An increase in self-efficacy and a decrease in parental stress were also associated with the parent–therapist alliance and the practitioners’ process and group management skills that promoted safe and supporting learning contexts [15].

Regarding the specific profile characterizing the professionals implementing evidence-based programs, Orte et al. (2014) asked a group of experts on a family-centered intervention program to identify the qualities of a good facilitator [20]. Through a Delphi technique, consensus was reached that facilitators should have, most of all, program training and family intervention experience, as well as good communication, empathy, and group management skills. However, this profile was developed only by academics and program facilitators, and the perceptions of parents were not included. Instead, parental experiences and perceptions of parenting programs have been mainly analyzed through qualitative research [8]. A systematic review of qualitative studies about general experiences with parenting programs revealed that parents value practitioners’ qualities, namely the ability to use a supportive and nonjudgmental approach, to instill hope, to model the techniques being taught, and to manage the dynamics within groups. Additionally, they value how practitioners facilitate relationships between parents and work with flexibility, allowing parents to contribute in terms of content without compromising the focus on the aims of the program [8]. In the specific case of parenting programs directed at child behavior problems, parents particularly value practitioners’ personal qualities (such as being non-judgmental, empathic and caring, flexible and adaptable, and collaborative, on one level with parents) along with the practitioners’ personal experiences (for example, being themselves the parent of a disruptive child) [21].

The role of the practitioner has been particularly emphasized in the Incredible Years (IY) Basic Parent Program [22], a well-known evidence-based parent group intervention for improving child behavior, proven to be effective in both prevention and treatment studies [23]. This intervention is largely similar in content and methods to other established multicomponent parenting programs based on the Hanf Model [24], although its strong emphasis on a collaborative group approach is a feature that distinguishes it from other parenting programs [22]. According to the program’s author [25], the implementation of this collaborative approach requires a considerable degree of clinical skills from the practitioner, perhaps even more than in other models, which makes this program ideal for the study of practitioner-related variables. Some studies with IY have already demonstrated that group leaders’ observed positive behaviors (such as praising, encouraging, or reflecting) predicted changes in positive parenting practices [13,26] or that parent-reported therapeutic alliance had a small although significant effect in reducing some children’s problem behaviors [27].

Less known, however, are the perceptions that parents have about the specific roles of IY practitioners in the context of this multicomponent intervention and the assessments that parents make of their own processes of change after the intervention has come to an end. Some authors consider that it is important to measure what individuals are saying to themselves about the change process [28] and that the picture of change is completed only by incorporating the first-hand accounts from intervention participants [29]. In the field of parenting interventions, quantitative studies have generally neglected parents’ perceptions about their own processes of change; instead, most of the existing evidence comes from the qualitative literature [8,29,30,31,32,33,34]. In fact, a few qualitative studies have tried to understand parents’ perceptions about the mechanisms of change of parenting interventions, and the role of the practitioner was often implicitly referred to, among other key factors of change such as program or group characteristics [29,30,31]. In fact, practitioners’ nonjudgmental support is frequently mentioned as a mechanism of change, as it encourages parental self-reflection and willingness to try out new parenting practices [30], while it also improves parental confidence, reducing feelings of guilt and isolation [32]. Moreover, the implementation of an experiential and collaborative approach allows parents to be involved in their own learning, which facilitates their engagement and their acquisition of new skills [32].

However, collecting the specific perceptions of parents through mixed-methods study designs may bring additional inputs to the perceived key mechanisms of change functioning in parent-based interventions, particularly to their knowledge about the assigned roles of the practitioners in the processes of change. A preliminary analysis undertaken by two of the authors used some of the present study’s data and focused on parents’ perceptions of the roles of different intervention factors on positive long-term changes following the IY intervention in a community setting [35]. In that study, parents recognized that the program’s contents and methods of delivery as well as the practitioners’ skills all contributed to their self-reported positive life changes. However, the specific role of the practitioners’ skills was not the focus of the study, and data on parents’ perceptions of the long-term changes were not cross checked with the change assessment data collected immediately after the intervention. In the current study, we deepened the analysis previously conducted [35] and used both quantitative and qualitative methods to analyze parents’ perceptions related to the practitioners’ specific roles in the IY intervention outcomes and their relationships to changes reported after the intervention. Specifically, the aims of the present study were: (1) to examine parents’ long-term perceptions of the impacts of the IY parent program on different aspects of their lives two years after enrollment in the intervention; (2) to analyze parents’ perceptions about the specific role of the practitioners delivering the intervention in promoting those life changes; and (3) to explore whether changes in parental sense of competence and in children’s behaviors, both immediately after the intervention, are related to parents’ perceptions about the role of the practitioners’ skills two years later.

## 2. Materials and Methods

**Figure 1:**
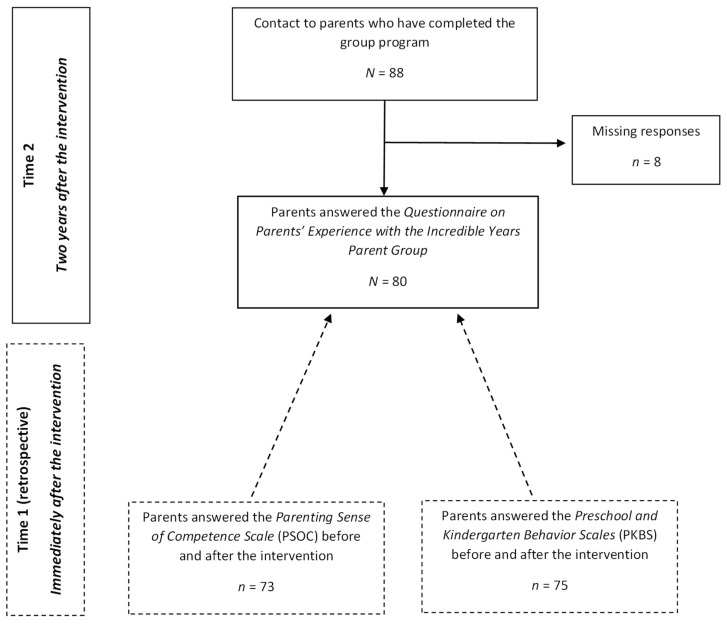
Flow chart of the participants’ assessment procedures.

This is a retrospective longitudinal study using data collected as part of an effectiveness trial testing the impact of the IY program when implemented by community providers in the context of Portuguese primary care services [36]. This article was partially based on data previously used [35], but we extended the focus to the practitioners’ skills and added two measures of change collected immediately after the intervention. In this study, two different assessment time points were considered: (1) immediately following the implementation of the IY intervention (Time 1) and (2) two years after the intervention (Time 2). Archival data were retrieved to assess changes in parents’ ratings of their sense of competence and in children’s behaviors (Time 1). Two years after the conclusion of the IY program, one survey was administered to measure parents’ perceptions about the life changes they experienced as a consequence of the intervention, as well as their perceptions of the roles of practitioners in enabling these changes (Time 2). Figure 1 illustrates this study’s longitudinal design and assessment procedures.

### 2.1. Procedures

Eighty-eight parents who had participated in the effectiveness trial [36] were invited by telephone to participate in the current study. We did not invite parents whose contact information was not made available (*n* = 8) or parents who had dropped out of the intervention (*n* = 6; we considered as dropouts those parents who attended four sessions or fewer in a total of 14 program sessions) because we were only interested in parents who completed the program; the point of this specificity was so that we could assess their perceptions about the specific roles of the practitioners’ skills and other components of the program in the life changes motivated by the intervention. After we explained the aims of the study and obtained agreement to participate from 88 parents, 80 returned the informed consent and completed the Questionnaire on Parents’ Experience with the Incredible Years Parent Group (response rate: 91%) at Time 2 (Figure 1). The eight missing parents had participated in seven different parent groups, and thus, missing participants were equally spread through different groups and practitioners. Archived data collected two years earlier were also retrieved for 73 of the participants in order to analyze changes in parental sense of competence and for 75 of the participants to analyze changes in their children’s behaviors assessed immediately at Time 1 after the intervention; seven and five participants, respectively, had missing responses at Time 1 in the measures of sense of competence and children’s behaviors. The study was previously authorized by the Portuguese Data Protection Authority (No. 1647/2016).

### 2.2. Participants

The participants in this study consisted of 80 parents of 67 preschool children who were voluntarily enrolled in a parent group with the IY Basic Parent Program within the context of an effectiveness trial [36]. Because this was a universal parent intervention, and despite some parents’ mention of concerns regarding their children’s externalizing behaviors in the recruitment interview, the only inclusion criterion in the study was having a child aged 3 to 6 years old. Participants were mostly married or living as married (94%) and had a mean age of 36.95 years (*SD* = 4.72) (Table 1). Of the 80 participants, 66 were mothers and 14 were fathers. While the majority of parents participated alone in the intervention (67.5% of the total sample and 68% of the married parents), there were also 13 couples. Half of the families had medium socioeconomic status (SES) as estimated by the parents’ occupation and years of education (based on the mean level of SES of both parents), and more than half (59%) had a university degree. Participants attended on average 11.94 of the 14 program sessions (*SD* = 1.90), with a minimum of 6 and a maximum of 14 sessions.

### 2.3. Intervention

The parental intervention given in the context of the effectiveness trial was founded on the implementation of the IY program at nine different primary care services in the central region of Portugal [36]. In brief, the intervention consisted of 14 weekly group sessions and two follow-up sessions, all of two hours each. The IY program’s contents focused on training parents in positive play and reinforcement skills aimed at increasing positive behaviors (such as child-directed play, descriptive commenting, praise and rewards), as well as a set of nonviolent discipline techniques aimed at reducing negative behaviors (including logical and natural consequences, ignoring, time-outs, and problem-solving strategies) [38]. Group discussions were facilitated by trained group leaders and relied on experiential techniques such as videotape modeling, behavioral rehearsal, and live modeling as key therapeutic methods [39].

The parent groups were led by 27 professionals, with 3 individuals working in teams and sharing the leading in each group. Each practitioner only facilitated 1 parent group during the study, and each group had an average of 11 participants (range: 9–12). The practitioners came from different professional backgrounds including psychology (*n* = 12), nursing (*n* = 7), early years education (*n* = 3), social work (*n* = 2), general medicine (*n* = 2), and speech therapy (*n* = 1). Although for 59% of the practitioners, this was their first experience as an IY group leader, they all had been trained in the intervention model via three days of training led by two certified mentors, and they all had attended at least two supervision sessions guided by credited peer coaches to support the implementation process.

### 2.4. Measures

#### 2.4.1. Questionnaire on Parents’ Experience with the Incredible Years Parent Group

This self-report questionnaire was developed by the authors for the present study in order to assess parents’ perceptions about the life changes they experienced after their participation in the IY intervention as well as the perceived role of the practitioner, along with other specific intervention components, in enabling those changes. The items on the questionnaire were developed based on the literature review and the existing IY measures (i.e., Satisfaction with the program’s questionnaire; Collaborative Process Checklist; and Peer and Self Evaluation Form) [40] and were adapted to the specific purpose of this study. The questionnaire was revised by five external IY professionals who have considerable experience in implementing and researching the IY program, and it was pretested with four parents who gave their opinions regarding the comprehensibility and relevance of the questionnaire’s items and structure.

The first section assessed the degree to which different areas of life had improved after the IY parent training (“Think about the impact that participating in the parent group has had in different areas of life…”). The response scale ranged from 1 (“It hasn’t improved at all”) to 5 (“It has greatly improved”). The remaining four sections assessed the degree to which three components of the intervention (program’s contents; program’s methods; practitioners’ role) and other factors of change external to the intervention had contributed to the life changes (“Please assess (…) the level by which each specific component has contributed to the identified changes in your life”). For these four sections, the response scales ranged from 1 (“It hasn’t contributed at all”) to 5 (“It has greatly contributed”). At the end of the questionnaire, two open-ended questions asked the subjects to describe if there were any other factors facilitating positive changes and any other factors that hindered changes.

In the present study, we mainly focused on the sections about the parents’ perceived life changes and the practitioner’s role in enabling those changes. The Perceived Changes section consisted of 10 items assessing the impacts of the parent program on different areas of life rated on the abovementioned five-point response scale. This section was analyzed considering the global score including all the 10 items (Cronbach’s α = 0.94) as well as the three different conceptual types of change defined a priori: changes in parental sense of competence, in parenting practices, and in parents’ cognitions (Parenting-related changes: α = 0.90; e.g., “My satisfaction as a parent”); changes in child’s behaviors and the child/parent relationship (Child-related changes: α = 0.82; e.g., “My child’s problem behaviors”); and changes in marital, family, or other interpersonal relationships (Interpersonal changes: α = 0.74; e.g., “The quality of my marital relationship”).

The Practitioner’s Role section comprised six items describing different practitioner skills (α = 0.93): (1) “The practitioners established a positive and authentic relationship with me”; (2) “The practitioners helped me to change my perspective, focusing on the positives, looking at the child’s perspective…”; (3) “The practitioners managed well the group dynamics and the organization of the session”; (4) “The practitioners shared their practical and technical knowledge in a clear way”; (5) “The practitioners were sensitive and flexible towards my needs, for example allowing me to bring my own questions to the sessions or adapting the sessions’ contents to the situations of my daily life”; (6). “The practitioners encouraged me to autonomously problem solve, for example encouraging me to look for solutions”). Each skill was assessed in two different ways: first, participants answered whether the practitioner had demonstrated the skill (Yes/No question), and then, they rated the extent to which the skill (if manifest) had a role in enabling life changes on a 5-point response scale (from “It hasn’t contributed at all” to “It has greatly contributed”). Finally, there was an open-ended question where parents could add other practitioner characteristics that they considered to have contributed to change.

The remaining sections of the questionnaire assessed the impacts of other intervention components and external factors on the parents’ perceived life changes. The Program’s Contents section comprised 11 items (α = 0.92) that included IY contents such as child-directed play and time-outs. The Program’s Methods included seven items (α = 0.89), such as role-plays or group and discussions in pairs during the sessions. The last section was about Other Factors of Change and consisted of five items (α = 0.82) assessing the roles of personal and child characteristics, social support, and other variables.

#### 2.4.2. Parenting Sense of Competence Scale

The Parenting Sense of Competence Scale (PSOC) [41] assesses parental perceptions of their competence as parents of children from 4 to 9 years old in 2 dimensions: Satisfaction (9 items; e.g., “Being a parent makes me tense and anxious.”) and Efficacy (7 items; e.g., “If anyone can find the answer to what is troubling my child, I am the one.”). Parents answer the questionnaire using a 5-point Likert scale (from “Strongly agree” to “Strongly disagree”). A total score for the 17 items was computed in this study, with higher scores indicating higher levels of confidence with respect to one’s own parenting capacities. In the present study, we computed a mean difference between the total scores obtained before and after the intervention to get a score reflecting the amount of change in PSOC evidenced after the intervention. The reliability in this sample for the total scale was 0.74 (pre-intervention) and 0.80 (post-intervention).

#### 2.4.3. Preschool and Kindergarten Behavior Scales

The Preschool and Kindergarten Behavior Scales—Second Edition (PKBS-2) [42] is an 80-item behavior rating scale designed to measure social skills and problem behaviors in children from 3 to 6 years old. For the present study, it was completed by parents. This instrument comprises 2 separate scales: a 34-item Social Skills Scale, for which a higher score indicates more skills, and a 46-item Problem Behaviors Scale, for which higher scores indicate more behavior problems. In the present study, we computed a mean difference between the subscale scores obtained before and after the intervention in order to obtain scores reflecting the amounts of change in social skills and in behavior problems after the intervention. In this sample, the reliability of the Social Skills scale was 0.91 (both pre- and post-intervention). The reliability of the Behavior Problems scale was 0.95 (also both pre- and post-intervention).

### 2.5. Data Analysis

Statistical analyses were performed using IBM SPSS 20.0. Prior to the data analysis, missing data were inspected. At the item level, missing values were random and were replaced by the respondent subscale mean if a maximum of 10% of items were missing. In the specific cases where a missing response had the meaning of not applicable (i.e., when the subject answered *No* to a *Yes/No* question about the presence of a specific skill and for that reason did not answer the subsequent question about the impact of that specific skill; *n* = 4), the missing value was replaced by the minimum value of the subscale (=1), meaning that the item/skill did not contribute at all to the perceived improvements. We also assessed whether there were differences between the participants from the present study and the parents who were invited to participate but did not return the questionnaire and who consequently were not included in this study. Comparative analysis between the two groups was made considering demographic characteristics, attendance at the intervention sessions, and changes in parental sense of competence and children’s behaviors reported immediately after the intervention, through Mann–Whitney and chi-square tests. Although there were 13 couples, data were analyzed for each parent individually because the aim of the study was to collect individual perceptions about personal experiences in the program; additionally, during the intervention program, each parent was also treated as an individual participant in the group.

To estimate changes in parental sense of competence, children’s problem behaviors and children’s social skills (Time 1), a mean difference was computed for each of these variables, subtracting the means obtained before the intervention from the means obtained post intervention; new variables were created accordingly. To explore parents’ perceptions about the changes derived from the IY intervention and the practitioner’s role in enabling those changes (Time 2), descriptive analyses were performed considering the frequency, mean (*M*) and standard deviation (*SD*), both of the items and the overall sections of the Questionnaire on Parents’ Experience with the IY Parent Group. In the Perceived Changes section, descriptive analyses were also performed for the three subscales (parenting-related, child-related and interpersonal changes). Paired t-tests were used to compare the mean of the practitioner’s role in change with the means of each of the other components of intervention (program’s contents and methods), as well as other factors of change. To examine whether the assessed changes in parental sense of competence and in children’s behaviors (Time 1) were associated with parents’ perceptions about the practitioners (Time 2), Pearson correlations were computed. To preliminarily explore parents’ responses to the open-ended question about additional practitioner skills that promoted change, we conducted a thematic qualitative analysis following the six recursive phases described by Braun and Clarke [43]: (1) familiarizing with data; (2) generating initial codes; (3) searching for themes; (4) reviewing themes; (5) naming themes; (6) reporting the results).

## 3. Results

### 3.1. Comparison between Participants and Parents Not Included in the Study

Parents who did not return the completed questionnaire (*n* = 8) did not differ significantly from the participants in terms of age, U = 317.50, *p* = 0.971, marital status, ꭓ^2^ (3, *N* = 88) = 0.66, *p* = 0.883, number of IY sessions attended, U = 299.5, *p* = 0.762, or assessed changes in children’s behavior problems, U = 233.5, *p* = 0.305, and social skills, U = 212.00, *p* = 0.174. However, those parents had lower SES, ꭓ^2^ (2, *N* = 88) = 8.71, *p* = 0.013 and lower academic qualifications, ꭓ^2^ (3, *N* = 88), *p* = 0.001 and reported a smaller change in their sense of competence, U = 128.5, *p* = 0.009, than did parents who were included in the study.

### 3.2. What Do Parents Perceive to Be the Impact of the IY Parent Program Two Years after Completing the Intervention?

Overall, participant parents perceived that many aspects of their lives had significantly improved because of the intervention in which they had enrolled two years before. As shown in Table 2, the mean of overall perceived changes for the 10 items was placed on the upper half of the positive range of the scale (*M* = 3.88, on a scale ranging 1 to 5). Most of the participants, 80%, gave the items average scores of 4 (*n* = 50) or 5 (*n* = 14), indicating a significant improvement in many aspects of their lives as a consequence of having enrolled in the intervention.

Regarding the specific changes identified in different areas of life, all the items presented means that were higher than the middle point of the scale, indicating that significant improvements were perceived in all the areas of life assessed. At the item level, the greatest changes were perceived in parents’ way of thinking about their children’s behavior (*M* = 4.21) and in child/parent relationship (*M* = 4.21), while the marital relationship was the area with the smallest perceived change (*M* = 3.28). At the subscale level, the Parenting-related changes were the most expressively reported (*M* = 4.07), followed by Child-related changes (*M* = 4.01).

### 3.3. Do Parents Perceive Practitioners to Have a Specific Role in Promoting Change?

Overall, parents perceived that the practitioners’ skills played a very important role in enabling their life changes related to their participation in the program (*M* = 4.24, range: 2–5). The perceived contribution of the practitioners’ skills was prominent, with a mean score that was significantly higher than the reported means for the roles of other intervention components, such as the program’s contents (*M* = 4.02, *SD* = 0.62, range: 2–5; *t*(79) = 4.38, *p* < 0.001) or methods (*M* = 3.93, *SD* = 0.68, range: 1–5; *t*(79) = 5.31, *p* < 0.001) or other factors of change (*M* = 3.74, *SD* = 0.68, range: 2–5; *t*(79) = 8.59, *p* < 0.001).

Regarding the specific practitioner’s skills (see Table 3), all parents reported that the practitioners evidenced the ability to establish a collaborative relationship with them, to effectively lead the group dynamics, and to manage the sessions as well as to share their knowledge in a clear way; 98.8% of parents reported that the leaders were skillful in changing their view and in promoting their autonomy, and 97.5% of parents recognized sensitivity and flexibility towards their needs. All skills had means above 4 (range 1–5), which did not allow us to identify meaningful differences between the practitioners’ skills scored by parents.

A brief and preliminary qualitative analysis of parents’ responses to the open-ended question on the questionnaire allowed for the identification of additional practitioners’ characteristics or actions that were also perceived as relevant to enabling changes. A thematic analysis [43] of the 19 responses written by parents identified 5 themes corresponding to 5 different practitioner’s skills. The skills most frequently reported by parents (*n* = 9) were Interpersonal relational skills, such as empathy, attentiveness, and acceptance without judgement, which promoted a close and familiar relationship between the practitioners and the parents, as well as a sense of security and trust that enabled parents to share without having the fear of being judged: “They were very attentive and human. A very intimate and harmonious group was created.”; “Thanks to the practitioners, I felt confident to share and supported by a group of ‘friends’ who are there to help without judging or pointing accusing fingers”.

The practitioners’ Dedication, commitment and availability to address parents’ doubts and difficulties whenever needed was also a reported characteristic (*n* = 7): “They were great, they were always available for anything we need, for our doubts and insecurities”; “Their availability during the program was wonderful and I felt that no question or doubt remained unanswered”. Some parents also underscored the importance of Objectivity and clarity in the problem-solving process (*n* = 4), as practitioners skillfully identified the problems and presented potential coping strategies in a calm, pragmatic and clear way: “They showed us different ways to deal with our problems in a calm, pragmatic and objective way”. Additionally, parents reported the relevance of practitioner’s Authenticity (*n* = 3), presenting her/himself with honesty and genuine enthusiasm, which facilitated a sense of trust (“I was surprised by the practitioners’ openness when introducing themselves, which allowed for that sense of trust”), and also the importance of Valuing each parent and reinforcing their sense of competence (*n* = 3) (“They valued my efforts as a mother”; “(…) [They] helped me to have more control in many situations with adults and children”).

### 3.4. Is Parents’ Evaluation of the Practitioners’ Skills Related to Previous Changes in Parental Sense of Competence and in Children’s Behaviors?

In order to examine whether there exists an association between the parents’ perceptions of the role of the practitioners’ skills and the changes reported two years before, immediately after the parenting intervention, in parental sense of competence (assessed by PSOC), children’s problem behaviors and children’s social skills (assessed by PKBS-2), we conducted two correlational analyses. In the first, we explored whether these change variables were associated with parents’ perceptions about the role of each component of the intervention in change (Table 4). The results showed that perceptions about the importance of practitioners’ skills (Time 2) were significantly associated with previously reported changes in children’s behaviors (in both problem behaviors and social skills) and in parental sense of competence (Time 1). The direction of the association suggests that the parents who reported the most improvements in parental sense of competence and in children’s behaviors immediately after the intervention attributed a greater role to the practitioners’ skills two years after the intervention. An improvement in children’s problem behaviors was also found to be associated with the importance assigned to the program’s methods, but no other component of the intervention had significant correlations with any of the change variables.

Additionally, correlational analyses were performed to explore the associations between the assessed change variables and the perceived role of specific practitioners’ skills (see Table 5). The results provided evidence that the practitioners’ sensitivity and flexibility were significantly associated with changes in all the domains assessed, i.e., parental sense of competence, children’s problem behaviors and children’s social skills; the ability to clearly share knowledge with parents was significantly related to improvements in children’s problem behaviors and social skills; the practitioners’ skill in terms of leadership and organization were associated with changes in children’s behavior problems; and the practitioners’ ability to promote parents’ autonomy and empowerment were significantly associated with reported improvements in children’s social skills.

## 4. Discussion

The present study analyzed the associations between parents’ perceptions about the practitioners’ skills and which different parent and family changes occurred after their participation in the Incredible Years (IY) parent intervention program. We specifically explored the role that parents assigned to the facilitators of the IY program in enabling long-term life changes two years after the end of the intervention, as well as the associations between parents’ retrospective evaluations of their practitioners’ skills and specific changes assessed immediately after the intervention. This study is particularly innovative as it focuses on parents’ perceptions about the intervention experience using a mixed-methods design, which allowed for the quantitative and qualitative exploration of the data. Our main findings indicated that two years after the intervention, parents perceived significant improvements, especially in their parenting and in their children’s behaviors, and they recognized that their IY practitioners had played a significant role in these life changes. Furthermore, we found that parents attributed a greater role to the practitioners’ skills than to other facets of the intervention, and those parents who attributed a greater role to the practitioners’ skills two years after the intervention also reported the most improvements in the parental sense of competence and in their children’s behaviors immediately after the intervention. The practitioners’ skills that related more broadly to these specific changes were the practitioners’ sensitivity and flexibility towards parents’ needs and their ability to clearly share knowledge with parents.

Overall, parents perceived that many aspects of their lives had greatly improved as a consequence of having enrolled in the IY program two years prior. Significant improvements were recognized, based on the mean scores, in the parenting domain (parental sense of competence, parenting practices and cognitions), in child’s behaviors and in the child/parent relationship, and finally in other interpersonal relationships (marital, family or other). Specifically, the greatest positive changes were perceived in the parents’ ways of thinking about their children’s behavior and in the parent/child relationship, and the smallest perceived change was in the marital relationship. These findings are aligned with previous research showing that the effects of parent training interventions directed at children’s externalizing behavior problems extend to several areas of life, although the effects are stronger in the areas explicitly targeted by the interventions; they are less expressive in domains more distal from parenting such as the quality of marital relationship [4,9]. However, it is important to consider that in our sample, 68% of married parents participated alone in the intervention, which may also help to explain why the improvements were less perceived in the interpersonal domain, particularly with regard to the marital relationship. In fact, research has shown that more impactful changes are experienced when both parents attend the intervention [44,45]. On the whole, however, evidence is mixed with respect to changes in marital relationship following a parent intervention, and there is some suggestion that the quality of couple relationship may be compromised if only one parent participates in the intervention [9].

When asked about the perceived role of practitioners in enabling the life changes related to their participation in the program, the parents assigned a prominent role to their practitioners’ skills. In fact, compared with the role assessment of other facets of the intervention (i.e., program’s contents and methods), the perceived overall contribution of the practitioners’ skills was significantly higher and related more broadly to improvements in children’s social skills, their behavior problems and parental sense of competence. Additionally, parents who attribute a greater role to the practitioners’ skills, two years after the intervention, had reported most improvements in parental sense of competence and in children’s behaviors, immediately after the intervention. Previous research within the field of parenting interventions has demonstrated that parents perceived the characteristics of their practitioners as a key factor of the intervention [8] and that they tended to focus more attention on therapist factors than on program factors [21]. Moreover, parents’ perceptions about higher levels of parent–professional alliance were significantly associated with improved clinical outcomes [15,46]. However, research has been sparse about the practitioners’ skills in evidence-based parenting programs and their relationships to the structured components of the implemented programs [15].

In the general field of family therapy, a great debate has arisen as to the specific and common factors in therapeutic change and whether the therapist is more important than the treatment itself [47]. More than taking this debate as a treatment *versus* therapist dispute, it is consensually assumed that it is only through the person and actions of the interventionists that every intervention works [48] and that even in manualized interventions, it is up to the practitioners to know what to do when and with what clients [49]. It is therefore understandable that from the intervention experience they had two years before, the participants in our study would particularly recall the practitioners’ skills and thus attribute them the main role in change. In fact, the IY practitioners are compared with “construction builders” ([50], p. 27) whose role is to rearrange all the components of the intervention so that they can be tailored to the individual family’s goals, culture, child temperament, child developmental level and family circumstances [39]. Therefore, they are the ones who activate, materialize and bring to life the components of the IY program, playing the main or more visible role in the intervention scene.

Regarding the practitioners’ specific skills more associated with change, our study demonstrated that there are some perceived practitioners’ skills that relate more significantly to changes in children’s behaviors and in parental sense of competence. These are, mainly, the practitioners’ sensitivity, flexibility and ability to clearly share knowledge with parents but also the practitioners’ skill of leadership and organization and their ability to promote parents’ autonomy and empowerment. The practitioners’ flexibility and sensitivity towards each parent’s specific needs is one of the fundamental principles guiding the IY program, which although manualized is not meant to be treated as a precise script or recipe to be recited to parents [39]. Several studies with different parenting programs have highlighted that parents valued a collaborative, non-directive approach to delivery, where strategies are suggested rather than taught and personalized or tailored to meet the specific needs of parents [8,33,34]. Thus, a balance between fidelity and flexibility seems to be required so that the practitioner can manage to “make the shoe fit” for each participant in the program ([29], p. 755). Our data reinforce this idea by showing that in the process of change, it is important that parents perceive their practitioners as being sensitive and flexible when implementing an intervention program.

The ability to share knowledge with parents in a clear way was also related to improvements in children’s problem behaviors and social skills, and it was highlighted in our qualitative analysis as well. Previous studies have mentioned the importance of clarity in practitioners’ communication [20]. In fact, increased awareness of children’s emotions and behaviors during the parenting program helps parents to adapt their practices [51], which makes sense in light of the fact that when parents better understand their children’s behaviors and the principles of childhood development, they are able to appreciate them differently and eventually to cope differently with them. In our qualitative analysis, clarity in communication was also related to objectivity and pragmatism in the problem-solving process. In fact, perceived clarity may also be associated with the practitioners’ skills in leadership and organization, which seem to be another important set of competences valued by the participants in this study. The IY program’s author underscores the importance of the group leader imposing structure in order to facilitate the group process [25]. A recent systematic review of parenting interventions concluded that parental change related to the therapist’s ability to structure the sessions, balancing between agenda and family goals [15].

Finally, the practitioners’ ability to promote parents’ autonomy and empowerment has been stressed in this study, in both quantitative and qualitative analysis. One of the stated roles of the IY practitioners is to empower parents through praise and validation, among other actions [50], and these competences are widely emphasized in the IY trainings. Previous quantitative research has suggested that parents’ sense of self-efficacy may be promoted by the non-judgmental support and collaborative approach from the IY practitioners [52] and that practitioners’ behaviors such as praising and encouraging predicted changes in positive parenting practices [13,26].

Three other specific practitioners’ skills perceived as relevant to enabling changes were added by some parents in the qualitative open-ended question, and these were: interpersonal relational skills such as empathy, attentiveness and acceptance without judgement; dedication and availability to address parents’ doubts and difficulties; and authenticity. Previous qualitative studies have already emphasized that parents value that practitioners are non-judgmental and non-patronizing, empathic and caring [21]. Authenticity has not been specifically referred to in previous studies with evidence-based parenting programs, but the IY program’s creator indicates genuineness and the use of self-disclosure as core conditions to positive and collaborative relationships with parents [25]. Moreover, previous research with parents of children with behavior problems has highlighted that practitioners’ personal dimensions are highly valued by parents [21], which suggests that more research can be developed on the topic of practitioners’ use of self and authenticity in the context of parenting programs’ implementation. It is interesting to note that all of the skills cited are generic therapeutic competences that contribute to the therapeutic alliance and are commonly required in any form of psychotherapeutic intervention [53]. The results of our study thus highlight some relevant practitioners’ skills that are both specific to the collaborative approach of the IY program and generic to all forms of therapeutic relationships. Together, our findings reinforce the idea that the practitioner’s assigned role when implementing a manualized and evidence-based parenting program goes far beyond the mere conveyance of the program’s specific contents and methods. The broad range of practitioners’ micro-skills may well constitute what Sanders and Burke named “the hidden technology of parent consultation” ([54], p. 1289). Although not in plain sight and scarcely studied so far, these practitioners’ skills may have an important and specific role in parental change, which indeed merits further research.

### Limitations and Directions for Future Research

This study has some limitations that should be noted. First, this is a non-randomized study that did not use a control group. As we were solely interested in understanding the perceptions surrounding the practitioners’ involvement in the intervention, we only used information from experimental groups. For this reason, this study does not claim to establish the efficacy and effectiveness of the IY program, as this has been previously done in other studies, but rather to explore parent perceptions about change and factors of change. There was an obvious homogeneity in parents’ responses, with the assessment of the practitioners’ skills being overall very high, which may have resulted in a “ceiling effect”. Parents may well have overestimated the role of the practitioners’ skills due to their overall satisfaction with the intervention, which is usually high [17,55]. However, our data also show that parents tended to rate practitioners’ skills in a more homogeneous and positive way than they rated other intervention components (e.g., program’s contents and methods), which stresses the specific importance that parents assign to practitioners. It is also possible that the measure’s items used to assess the role of the practitioners’ skills were not specific enough. In fact, the measure used in the current study to assess parents’ perceptions about the practitioners included composite items describing practitioners’ macro-skills, while there is some suggestion that assessing more microlevel simple behaviors and skills may be more informative [15,56] and may lead to more variability in the data. The fact that we were examining the memories of parents about the practitioners’ skills demonstrated almost two years prior prevented us from using a measure assessing practitioners’ micro skills and behaviors, as it was easier for our participants to recall more generic skills. We presented in the Method section a detailed description of the items included, as well as their internal consistency. It also bears taking into account that parents assessed the skills of their group leaders conjointly, which increased the likelihood of having observed the skills. In future studies, however, it would be valuable to include measures specifically developed to assess each practitioner’s skills with more detail and thoroughness and to use them at different assessment times (e.g., immediately after the intervention).

In fact, another limitation to consider is the fact that respondents are recalling memories from an intervention that took place two years prior, which attenuates their ability to distinguish between different practitioners’ skills and which may impact the reliability of their responses. In addition, parents’ perceptions may have been influenced by different life events occurring in the period after the intervention. In an attempt to control this variable, parents were asked to respond to a small survey about specific life events occurring during this follow-up period. However, since only 19 parents (24% of the sample) answered this survey, it was not possible to use this data in the analysis. Therefore, we underscore that the main goal of the present study was to understand parents’ long-term perceptions about the intervention-related factors of change and not the real effectiveness of the program. We have tried to complement the analyses using measures collected immediately after the intervention and have also explored the qualitative responses asking for additional relevant practitioners’ skills. Given the small number of parents answering the open-ended question, the qualitative part of this study is only preliminary and does not allow for the construction of new theory. However, the informative richness of the qualitative answers suggests that there is room for much more exploration on the subject of the specific practitioners’ skills perceived as contributing to positive outcomes, pointing to a need for more qualitative research in this field. Indeed, qualitative studies may be of special value in order to better understand the specific role of the practitioner in parental and family change and eventually to inform the development of new measures of practitioners’ skills.

The small and homogeneous sample used in this study presents an impediment to the possibility of any firm generalization of the findings. Participants were mostly educated mothers, with a medium-high socioeconomic status, who volunteered within a community context. With a larger and more diversified sample, it would be interesting to explore the potential moderator role of variables such as parents’ gender (fathers/mothers) and SES, whether an individual is participating together with a co-parent, and the child’s characteristics (clinical/non-clinical). The fact that the parents who did not respond to the questionnaire developed for our study had some socioeconomic differences and reported less change in parental sense of competence than the study’s participants is another limitation to consider in the interpretation of our findings. Although non-respondents are a minority (only 9% of the total of contacted parents), these findings suggest that our results may not be generalizable to all parents and should account for parents’ level of education, academic qualifications or sense of competence felt after the intervention. In the context of increasing diversity in the parenting role, future research should strive to include more diverse parents, particularly those who are less reachable and who might have perceived fewer changes following the intervention. These parents’ perceptions about the practitioners’ role in the intervention may contribute to additional insights on this topic.

## 5. Conclusions

Our results expand on the existing literature about the prominent role of the practitioners in an evidence-based parenting program and have implications for the field. Specifically with respect to the IY program implementation, our results reinforce the evidence that practitioners from different professional backgrounds and with varied experience with the program may exhibit an overall sufficient level of skills proficiency (as perceived by parents), which suggests that the IY model of training and supervision may be adequate to assure the quality of its delivery. In fact, the IY model has always emphasized the primary importance of practitioners’ skills and has developed a well-structured process to support the professional development of the IY leaders, with a great emphasis on on-going training, supervision and coaching [57]. Given the level of sophistication of the practitioners’ skills required in this field, other parenting programs should implement similar processes to support the continued development of their practitioners. This study also reinforces that the support and monitoring of the professionals should not only be a concern during the training periods but also a continued priority over time, through services that are easily accessible in community contexts. Particular attention may be required for the development of practitioners’ sensitivity to parents’ personal needs and flexibility to adapt program’s contents to the characteristics of each family, as these skills were specially highlighted in the results of our study. Finally, in order to better adjust these interventions according to families’ actual needs, greater efforts may be required to collect parents’ perceptions about their own processes of change and specifically about the practitioners’ skills that they value the most.

## Figures and Tables

**Table 1:** Participants’ Characteristics.

Characteristics	*N*	%
Relationship with the child		
Mothers	66	82
Fathers	14	18
Marital Status		
Married/as married	75	94
Divorced/Separated	1	1
Single	4	5
Family SES ^a^		
Low	13	19
Medium	34	50
High	21	31
Academic Qualifications		
6th–9th grade	8	10
High school	11	14
Graduation	47	59
Master/PhD	14	18
Participation in the group		
Solo	54	67.5
In couple	26	32.5

^a^ SES was defined using a classification developed for the Portuguese population considering three categories [37]: low (unskilled workers, e.g., industry, transport, agriculture, and fishery workers); medium (intermediate technicians, e.g., administrative, trade and services professionals); and high (e.g., owners and entrepreneurs, managers, scientific and intellectual professionals).

**Table 2:** Frequencies and Mean Values of the Perceived Changes.

Specific Changes Subscales	Perceived Changes—Items	Frequencies of Responses to Items (%)					Item Mean (*SD*)	Subscale Mean (*SD*)
		1	2	3	4	5		
Parenting-related changes	1. Job as a parent	0	0	17.5	62.5	20	4.02 (0.62)	4.07 (0.62)
	2. Satisfaction as a parent	1.2	1.2	15	58.8	23.8	4.02 (0.75)	
	3. Way of thinking about the child behavior	0	0	11.3	56.2	32.5	4.21 (0.63)	
	4. Way of acting with the child	0	5	16.3	50	28.7	4.03 (0.81)	
Child-related changes	5. Child’s positive behaviors and social skills	1.2	2.5	16.3	60	20	3.95 (0.76)	4.01 (0.62)
	6. Child’s problem behaviors	0	2.5	27.5	50	20	3.88 (0.75)	
	7. Child/parent relationship	0	1.2	8.8	57.5	32.5	4.21 (0.65)	
Interpersonal changes	8. Marital relationship	11.2	5	37.5	37.5	8.8	3.28 (1.08)	3.49 (0.74)
	9. Family relationships	2.5	5	33.8	45	13.7	3.63 (0.88)	
	10. Other relationships	1.2	3.8	40	47.5	7.5	3.56 (0.74)	
Perceived Changes overall Mean (*SD*)								3.88 (0.59)

**Table 3:** Practitioners’ Skills that Enabled Parents’ Change.

Practitioners’ Skills			How Much Did the Skill Enable Change?							
	Was the Skill Present?		Frequency of Responses to Items (%)					Min	Max	Mean (*SD*)
	Yes	No	1	2	3	4	5			
Collaborative relationship	100.0	0.0	0.0	1.2	6.3	56.2	36.3	2	5	4.28 (0.64)
Changing perspective	98.8	1.2	1.2	1.2	3.8	55.0	38.8	1	5	4.29 (0.72)
Group leadership	100.0	0.0	1.2	1.2	10.0	50.0	37.5	1	5	4.21 (0.78)
Sharing knowledge	100.0	0.0	1.2	0.0	7.5	51.3	40.0	1	5	4.29 (0.72)
Sensitivity/Flexibility	97.5	2.5	1.2	1.2	11.3	52.5	33.8	1	5	4.16 (0.77)
Promoting autonomy	98.8	1.2	1.2	1.2	12.5	46.2	38.8	1	5	4.20 (0.80)
Practitioner’s role overall mean										4.24 (0.64)

**Table 4:** Correlation coefficients between the perceived role of different components of the IY intervention and assessed changes in parental sense of competence and in children’s behaviors.

	Change in Parental Sense of Competence	Change in Children’s Problem Behaviors	Change in Children’s Social Skills
Program’s contents	0.09	−0.16	0.14
Program’s methods	0.22	−0.26 **	0.20
Practitioners’ skills	0.23 *	−0.25 **	0.28 **

* *p* = 0.05, ** *p* < 0.01.

**Table 5:** Correlation coefficients between specific perceived Practitioner’s Skills and assessed changes in parental sense of competence and in children’s behaviors.

	Change in Parental Sense of Competence	Change in Children’s Problem Behaviors	Change in Children’s Social Skills
Collaborative relationship	0.19	−0.15	0.21
Changing perspective	0.18	−0.10	0.21
Group leadership	0.22	−0.27 *	0.18
Sharing knowledge	0.19	−0.26 *	0.30 **
Sensitivity/Flexibility	0.30 *	−0.28 *	0.29 *
Promoting autonomy	0.13	−0.22	0.28 *

* *p* < 0.05, ** *p* < 0.01.

## Data Availability

The data presented in this study are available on request from the corresponding author.
